# Suicidality in University Students Throughout the COVID-19 Pandemic

**DOI:** 10.1192/j.eurpsy.2022.830

**Published:** 2022-09-01

**Authors:** D. Vigo

**Affiliations:** University of British Caolumbia, Psychiatry, Vancouver, Canada

**Keywords:** students, Suicide, mental health, Covid-19

## Abstract

**Introduction:**

The COVID-19 pandemic has greatly disrupted the day-to-day life of university students, as it has for the general population. University students have been reported to have high rates of mental health concerns, including suicidal ideation.

**Objectives:**

Ascertaining the correlation of Covid-19 dissemination and proximity to University students in Vancouver, Canada, with suicidal ideation and suicidal plan.

**Methods:**

We analyuzed weekly cross-sectional data from our Canadian World Mental Health International College Student survey by plotting the 30-day suicide ideation as a binary and the ordered 30-day suicide ideation outcomes using logistic and ordered generalized additive model (GAM) respectively, with a cubic spline and adjusting for demographics. We also ran an analysis on the association between binary 30-day ideation and different sample characteristics using logistic regression.

**Results:**

The time trend analysis showed that suicidal ideation did not seem to increase during the COVID-19 pandemic. On the contrary, ideation levels were found to be high in the beginning (February 2020) with a downwards trend through June to September before gradually increasing around November, 2020. We identified sociodemographic risk factors that may be associated with suicidal ideation, and established that those most at risk were students who had been emotionally overwhelmed by Covid-19 and unable to find help.

**Conclusions:**

Our results seem to indicate that, in general, students have remained resilient under the stress factors presented by the pandemic, and that trends in suicidality seem to follow seasonal or school calendar year stressors rather than respond to the pandemic. However, certain subpopulations appear to be more affected than others.

**Disclosure:**

No significant relationships.

